# Should I stay or should I go? DNA damage thresholds drive stay-or-go decisions in *Acinetobacter baumannii* biofilms

**DOI:** 10.1128/jb.00545-25

**Published:** 2026-07-20

**Authors:** Brian Nguyen, Veronica Godoy-Carter

**Affiliations:** 1 Department of Biology, Northeastern University 198849, Boston, Massachusetts, USA; University of Southern California, Los Angeles, USA

**Keywords:** DNA damage response (DDR), biofilms, *Acinetobacter baumannii*, RecA

## Abstract

*Acinetobacter baumannii* is a clinically significant pathogen that is difficult to eliminate from infected individuals; indeed, infections due to this organism cost the health care system tens of millions of dollars per year. This pathogen readily infects immunocompromised individuals and can survive extreme conditions, including the osmotic and immune stresses of the human host and prolonged desiccation on hospital surfaces. The survival success of *A. baumannii* is in large part due to the formation of multicellular communities, or biofilms, that afford cells protection from external stresses, such as antibiotics and desiccation. Cells within biofilms cycle between a surface-attached, protected state and dispersal, in which they egress the biofilm and disseminate to new niches while becoming vulnerable to antibiotics and host immunity. The fate decision to either stay or leave the biofilm is just beginning to be understood in *A. baumannii*, and we review the current state of knowledge here. Emerging evidence suggests that the DNA damage response (DDR), and specifically the RecA protein, is key to orchestrating this decision.

## ACINETOBACTER BAUMANNII—A NOSOCOMIAL INFECTION THREAT

The *Acinetobacter* genus belongs to the Moraxellaceae family*,* differing from other members by being catalase-positive and oxidase-negative. *Acinetobacter* spp. members are gram-negative, aerobic, nonfermenting, nonfastidious bacteria that move by twitching and, under some conditions, by swarming (1–5). *Acinetobacter baumannii* has a coccobacilli shape, with an approximate size of 1.0–1.5 µm by 1.5–2.5 µm during the exponential phase and becomes more cocci-shaped in the stationary phase. *A. baumannii* was initially characterized in 1968 (6), though it was formally classified only in 1986 (7, 8). A detailed story of the Acinetobacter genus and *A. baumannii* taxonomy has been recently reviewed (9).

Initially, *A. baumannii* was known as Iraqibacter; soldiers from the Iraq war came back stateside with troublesome infections involving a then-unknown gram-negative bacterium that gained antibiotic resistance quickly (10–14). Since then, *A. baumannii* has become a major cause of hospital-acquired infections around the world. It is part of the ESKAPE group of pathogens, which include *Enterococcus faecium*, *Staphylococcus aureus*, *Klebsiella pneumoniae*, *Acinetobacter baumannii*, *Pseudomonas aeruginosa*, and *Enterobacter* species; pathogens that make up most of the antibiotic-resistant hospital-borne infections (15–17). Nosocomial infections caused by *A. baumannii* include infections in the skin and soft tissue, burns, urinary tract, and meningitis, though the most serious infections are ventilator-associated pneumonia and bloodstream infections (18–21). *A. baumannii* infections are the leading cause of mortality rates in intensive care units, especially those caused by carbapenem-resistant *A. baumannii* infections (22–24). Crude mortality rates for pneumonia range from 40% to 70%, while mortality rates for bloodstream infections range from 28% to 43% (18). Community-acquired *A. baumannii* has also been reported (25–27). These are associated with individuals with risk factors such as alcoholism, smoking, diabetes mellitus, and chronic respiratory illnesses (28, 29). The source of the infections is unknown, but there is a correlation with humid and hot weather climates (29). A study comparing the two types of infections found that community-acquired infections had a higher mortality rate due to misdiagnosis in intensive care units (57.8% vs 35.4%, respectively) and a much shorter median survival time (8 days vs 103 days) (30).

In summary, *A. baumannii* is a pathogen not only capable of persisting in patients but also of surviving extreme conditions such as desiccation (31), while readily acquiring resistance to multiple antibiotics (11, 12, 20, 21, 32–35). The Centers for Disease Control (CDC) has noted that, in the US, over 60% of infections are multidrug-resistant (36). Increasing antibiotic resistance, namely resistance to carbapenems (22, 37–39), has led the CDC to classify *A. baumannii* as an “urgent” threat, its highest classification (Centers for Disease Control, 2019).

*A. baumannii* survives extreme conditions mostly thanks to its ability to form biofilms, which will be discussed below.

## PROFILE OF THE BIOFILM CYCLE IN *A. BAUMANNII*

Biofilms are understood as multicellular communities of bacteria that form a self-produced protective extracellular matrix composed of DNA, sugars, and proteins (40–43). Most bacteria survive in the environment and in infected individuals as biofilms (43–49). Bacterial cells go through a cycle of biofilm formation and dispersal (41, 50, 51), making cell fate decisions that are mediated by global transcription factors. Biofilm formation is generally better understood than dispersal (52–54). This gap is especially pronounced in *A. baumannii*, where the mechanisms governing dispersal remain poorly characterized. Compounding this challenge, *A. baumannii* lacks flagella (55, 56), meaning it cannot actively swim away from a surface, raising the fundamental question of how dispersal is physically achieved and regulated in this organism.

A two-component regulatory system, *bfmRS*, enables biofilm formation in *A. baumannii* (57–62). BfmS is the membrane-bound sensor kinase protein that phosphorylates the transcription factor BfmR at the D58 residue (D, Aspartic acid) (58, 63). Phosphorylated BfmR (BfmR-p) forms a homodimer and becomes a strong DNA binder, negatively regulating itself (63). ∆*bfmR* strains do not form biofilms (57–62, 64), while unexpectedly, ∆*bfmS* strains show high BfmR-p and better survival in the host (63, 65, 66). A recent study identifying the DNA sequences recognized by BfmR using a genome-wide ChIP-Seq experiment (63) showed that BfmR activates or represses genes involved in cell wall structure, capsule, and outer membrane biosynthesis, as well as genes that encode various inner membrane proteins and the Type IV pili responsible for twitching motility (63). Loss of motility is a signature of cells in biofilms (67, 68), and this is accomplished in *A. baumannii* by BfmRS-mediated repression of components of the Type IV pili (63). The signaling required to induce BfmR phosphorylation remains unknown, though exposure to certain antibiotics results in the hyperproduction of K-locus capsular polysaccharides, a *bfmRS*-dependent gene (69).

Among the genes activated by *bfmRS* are OmpA, which enables adherence to epithelial cells and survival in human serum (59, 65, 70). OmpA has similar roles in other bacteria, such as *Escherichia coli* and *Pseudomonas aeruginosa* (71, 72). In agreement with the ChIP-Seq data (63), an *ompA*-deficient strain in *A. baumannii* showed biofilm reduction and lower attachment to eukaryotic cells (65, 73). The Csu pili is another of the well-known attachment pili in *A. baumannii* (74–81) (see section below) that requires BfmRS (62), but there is no direct binding of BfmR to the promoter of its gene (63).

Biofilm maturation is another step in the formation of biofilms (68). In *A. baumannii,* proteins needed for the organization of biofilm structure (82, 83) are secreted *by the A. baumannii* type I secretion system. An example is the biofilm-associated protein (Bap) (82, 83), an acidic (pI ~3), large molecular weight, cell surface protein (82) with repeated units like the one originally discovered in *Staphylococcus aureus* (84–86). The *S. aureus* Bap protein forms amyloid filamentous structures that organize biofilm structure (84), like the Tas protein in *Bacillus subtilis* (87, 88), as loss of Bap reduces biofilm thickness and volume (89). Bap is important for biofilm maturation and stability (83) and for cell-surface adhesion to both biotic and abiotic surfaces (90, 91). It is unclear how *A. baumannii* Bap protein expression is regulated, as well as its mechanistic relationship, if any, to the *bfmRS* regulon.

The efflux pump, AdeIJK, involved in multidrug resistance (15, 92–94), has also been identified as an important factor in biofilm maturation since it releases biosurfactants (89), and its overexpression in clinical strains correlates with robust biofilms (94). Additional gene products, such as Lon protease, play a role in biofilms, but the mechanism is unclear (95). Other factors that have been identified as important for biofilm formation include poly-β-(1-6)-*N*-acetylglucosamine (96) and extracellular DNA (97). For additional details, see a recent review by Ayswarya et al. (89).

To enable biofilm dispersal, existing regulatory pathways may be reversed, or entirely new ones may emerge. The link between the DNA damage response (DDR) and the biofilm cycle in *A. baumannii* illustrates both possibilities (96, 97). The mechanistic details of the DDR in most bacteria have been previously summarized and heavily studied (98–112). To gain insights into the biofilm-DDR axis, we will describe in the sections below the knowledge gained in the regulation and main transcription factors known to play a role in one or both processes.

## A BRIEFING ON THE DDR IN *A. BAUMANNII*

Unlike other DDRs (102), the *Acinetobacter* genus lacks a LexA homolog and classical DDR genes do not share a common regulator, and thus no common regulator DNA sequence binding motif (99, 113, 114). Due to this, it was initially believed that *A. baumannii* did not have a coordinated DDR (114). In fact, the DDR is highly dependent on *recA* expression (99).

*A. baumannii* has an inducible DDR, which includes canonical DDR genes (99). Indeed, increased mutagenesis and antibiotic resistance are dependent on the expression of *recA* and ultimately error-prone or translesion synthesis (TLS) DNA polymerases, such as DNA Polymerase V (99).

**Fig 1:**
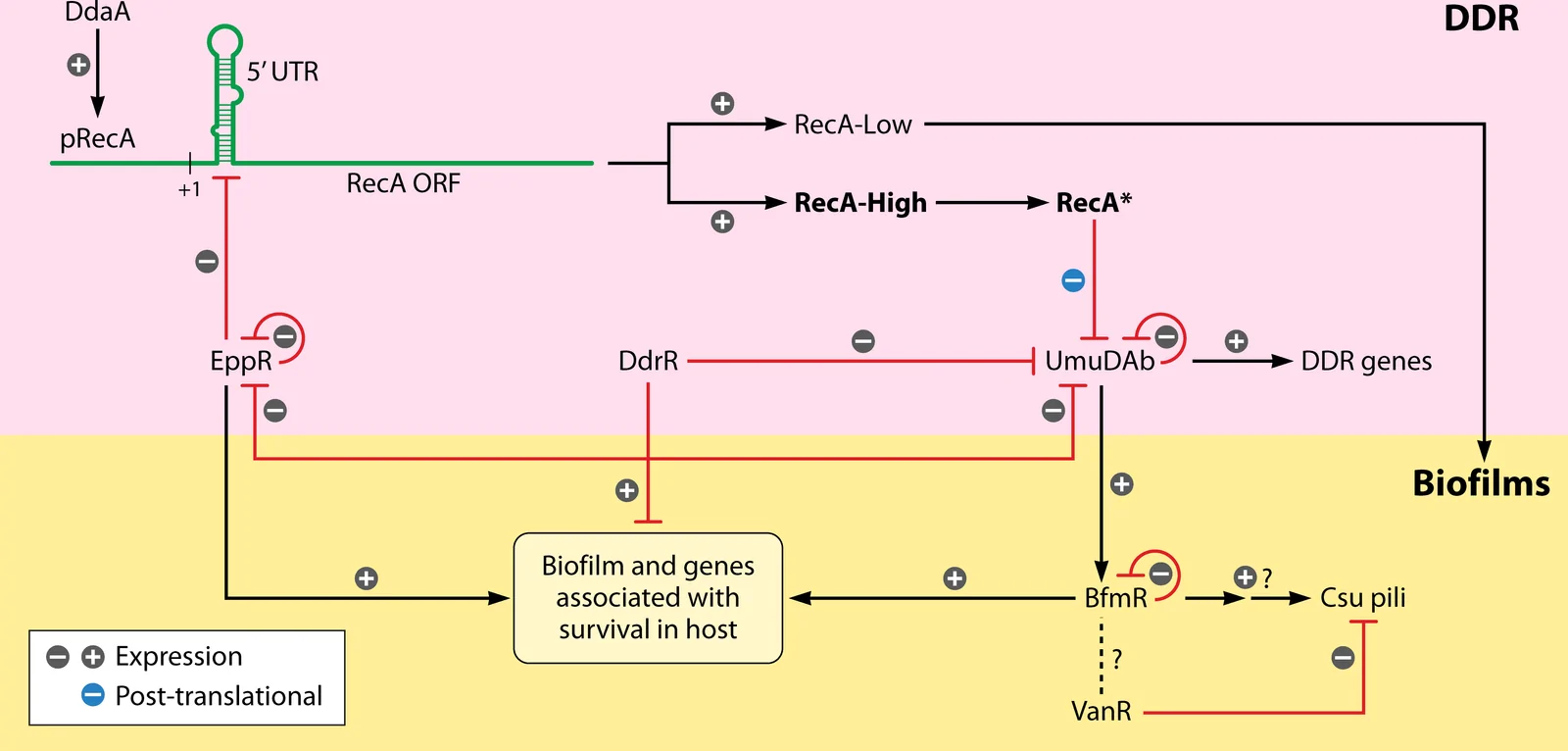
DNA damage and biofilm regulation in *A. baumannii.* Shown are genes we know are both part of the DDR and of biofilms and their regulation. The genes are described in detail in the text. DdaA activation of *recA* gene expression was recently described (117), while RecA is at the center of DDR regulation (99) and the formation of DDR subpopulations (115). The recA 5′UTR enables formation of RecA subpopulations (118) and likely of other DDR genes. UmuDAb is a DDR repressor defined by transcriptomics and abundant genetic data (113, 115, 119). The evidence about the connection between the DDR and biofilms, the biofilm-DDR axis, has been provided by us (120). BfmR-p is the master biofilm regulator, acting as an activator and repressor of biofilm cycle genes (63) and genes associated with *A. baumannii* survival in the host (63). BfmR is phosphorylated by BfmS, the sensor kinase, becoming BfmR-p homodimer, enabling robust DNA binding (63). The signal recognized by BfmS is unknown. BfmR indirectly induces one of the main attachment pili, Csu. It is VanR, a repressor of vanillin catabolism, that directly represses Csu expression (121). Most of the known transcription factors involved in the DDR are also involved in biofilms. The section of the diagram in pink represents DDR genes, while the yellow represents biofilms. Low RecA is shown to direct biofilm formation; R, resistance.

In response to DNA damage, *A. baumannii* forms two distinct phenotypic subpopulations, one where cells highly express RecA and other DDR genes, and the other where there is low expression (115) (Fig. 1). As expected, cells with high expression of RecA and the DDR are more mutagenic (99), and they survive DNA damage treatment (115), but seem to eventually suffer error catastrophe (116), as a large fraction of them die after DNA damage treatment is lifted. While cells with low RecA and DDR expression have higher overall survival (115).

This heterogeneous DDR response is a bet-hedging strategy for survival and provides genomic plasticity. A structured untranslated region (UTR) on the 5′ end of the *recA* transcript controls phenotypic variation (see below for more details) (118). However, the transducing mechanism between RecA phenotypic variation and other DDR genes remains unclear.

The *A. baumannii* DDR also includes UmuDAb, a transcriptional repressor (98, 115, 119, 122, 123) and an activator of DDR (98) and DDR-independent genes (120). In response to DNA damage, RecA becomes RecA*, as in the canonical SOS (124), leading to UmuDAb autocleavage and derepression of genes encoding DNA Pol V (122, 125).

A recent RNAseq study described an additional possible transcription factor, DdrR, involved in the regulation of TLS DNA polymerases with UmuDAb. The DdrR transcriptome suggests that the regulatory role of DdrR extends beyond DDRs and includes genes involved in many different processes such as translation, metabolism, quorum sensing, biofilm formation, and secretion systems (113, 123, 126–129)

In short, the diagram in Fig. 1 shows transcription factors and genes involved in both biofilms and the DDR. RecA is at the center of both processes. Thus, any transcription factor that regulates *recA* affects *A. baumannii* cell fate (reflected as cells within a biofilm or free living) and the DDR. The evidence is described below.

## REGULATION OF *A. BAUMANNII RECA* EXPRESSION; AN ACTIVATOR AND A REPRESSOR

DdaA activates (117), and EppR represses *recA* expression by, respectively, binding directly to a motif in the *recA* promoter and to the DNA sequence transcribed into the 5′UTR of the *recA* mRNA (130) (Fig. 1). EppR is a TetR repressor (TNF), which has a well-conserved helix-turn-helix DNA binding motif and a sensor domain (131, 132). The expression of the genes that TNFs regulate depends on the binding of a ligand to the sensor domain (133), which results in a conformational change that abolishes (or permits) DNA binding, and gene expression is either induced or repressed (134). EppR is the first TNF associated with the regulation of the DDR. This finding suggests that EppR binds a yet unknown ligand that is accumulated because of DNA damage, the first of its kind (130).

It is not yet known how or if the *recA* activator, DdaA, is regulated. We know that the *recA* repressor, EppR, forms a homodimer and represses itself and the genes encoding DNA Pol Vs, in addition to UmuDAb (130). Moreover, evidence suggests the intracellular concentration of EppR is low (130), which may explain why RecA levels are relatively high at basal levels (115). Possibly, this is the condition in which there is low DNA damage, and DdaA is not activating *recA* expression. When cells are treated with DNA-damaging agents such as Ciprofloxacin (135), RecA-induced levels are ~4-fold higher than basal, much lower than the ~10× induction in *E. coli* (120). We can infer that the intracellular RecA levels in *A. baumannii* are maintained such that cell fate and mutagenesis (or viability) are kept at a level that allows for survival. In summary, *recA* expression is maintained by an activator and a repressor that in turn affect the cell's fate (Fig. 1). Finally, *recA* regulation also includes a 5′UTR responsible for cells within a population to have high and low RecA levels with their distinct cell fates (Fig. 1).

## THE *RECA* 5′UTR ENDOWS *A. BAUMANNII* WITH PHENOTYPIC VARIATION

**Fig 2:**
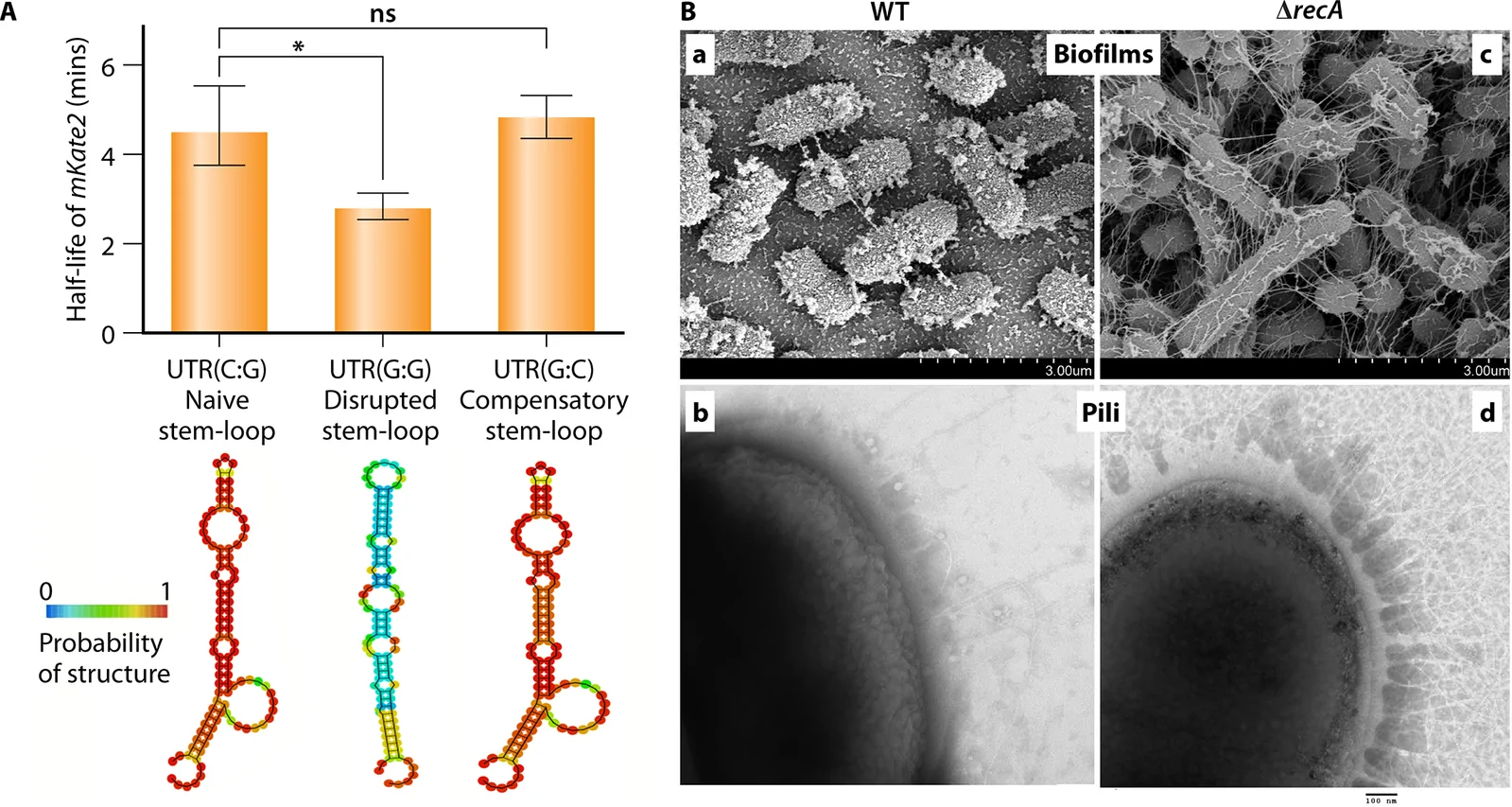
RecA subpopulations and biofilms depend on the mRNA stability provided by its structured 5′UTR. The main recombinase RecA’s transcript is stabilized by a 5′UTR. (**A**) The half-life of mKate2 was measured when fused to the different predicted UTR derivatives shown below. The native UTR structure endows the mRNA with a long half-life that is significantly decreased by a disrupted UTR. The mRNA stability is recovered by compensatory mutations in the UTR. The probability of the UTR structure is shown on the left-hand side, with the predicted UTRs shown to the right of it. Taken and modified from supplementary data (118). (**B**) The *A. baumannii* ∆*recA* strain forms robust biofilms and displays more pili. (a and b) Scanning and transmission electron micrographs of 24 h pellicles formed by *A. baumannii* ATCC17978 (WT) show biofilms and pili. (c and d) The ∆*recA* strain displays more biofilms and pili on its surface. Modified from Ching et al. (120). Size bars are 3.0 µm for biofilms and 100 nm for pili. Micrographs taken by V.G. and P. Muller.

A protein fusion of the *recA* 5′UTR to a fluorescent reporter showed that there were two RecA cell subpopulations, one with high and one with low RecA (120). In this case, the *structured* 5′UTR promotes RecA translation, while the linear UTR or its absence does not (118) (diagram in Fig. 1 and 2A, native stem-loop). Therefore, its function is different from those of well-known ribosensors/riboswitches, in which the structured 5′UTR/aptamer inhibits, while the relaxed form permits gene expression (136, 137). The 5′UTR is conserved in all the *recA* gene sequences we have looked at in *A. baumannii*, but not in other Acinetobacter such as *A. baylyii* (115, 118).

When the stability of the native *recA* transcript and that of various UTR derivatives with predicted distinct stabilities was measured, it was found that the *recA* mRNA with the native structured 5′UTR is at least four times more stable than the others (Fig. 2A) (118). Thus, the simplest explanation is that mRNA stability endowed by the structured 5′UTR promotes better RecA translation and high levels of RecA. We believe this is not the whole story and are working to elucidate this mechanism. These data suggest that the 5′UTR provides a fitness advantage and may explain why RecA levels are carefully orchestrated in *A. baumannii*. The goal is to promote fitness and avoid killing of the entire population.

## RECA LEVELS ORCHESTRATE THE BIOFILM CYCLE IN *A. BAUMANNII*

**Fig 3:**
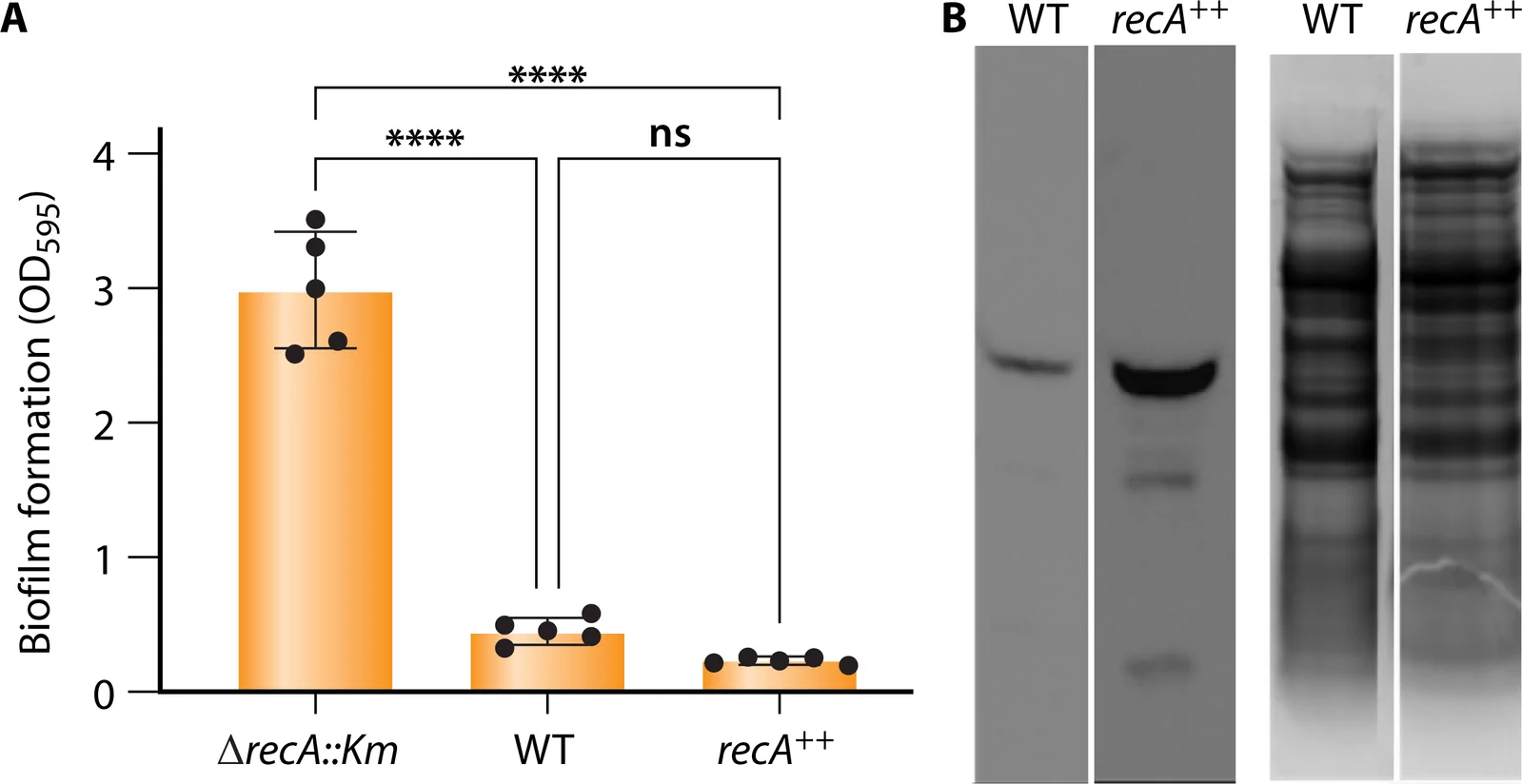
RecA levels and biofilms. (**A and B**) Quantification of biofilms formed by the strains shown. ∆*recA* forms significantly stronger biofilms than the WT and a strain with excess RecA (*recA*^++^). **** indicates significance *P* < 0.0001, while “ns” is not significant. Modified from Ching et al. (120). A western blot modified from supplementary material in Ching et al. (120) demonstrates that the *recA*^++^ strain has more RecA than the WT. The levels of the *recA*^++^ strain are similar to those of fully induced RecA in response to DNA damage (120).

When studying the *recA* regulation by the 5′UTR, we came upon a striking revelation; the ∆*recA* strain showed robust biofilm formation (120) (Fig. 2B and 3A). This phenomenon is similar to the one observed in *Bacillus subtilis*, which we published in collaboration with the Chai Lab (138). When there is elevated DNA damage within the *B. subtilis* biofilms, cells escape to find new niches to settle and start the cycle again. The same concept is true for *A. baumannii* (Fig. 3A), though in this case, it is directly related to RecA levels (Fig. 3).

In conditions of low/no DNA damage, Low RecA/no RecA keeps UmuDAb intact, while High RecA would result in UmuDAb cleavage thanks to the formation of RecA*, promoting its cleavage in a reaction like LexA (108) (diagrammed in Fig. 1).

The evidence suggests that cells stay in biofilms when there is no/low DNA damage, and the opposite occurs at high DNA-damaging conditions. In fact, high RecA promotes biofilm dispersal as shown by confocal microscopy, where the thickness of the biofilm pellicle was measured after treatment with sublethal concentrations of ciprofloxacin (120) known to induce the DDR (115). Dispersed cells are endowed with high mutagenic potential and can become antibiotic resistant, though they are susceptible to antibiotics and any possible host defense since they are no longer protected by the biofilms. Cells leaving the biofilms would be able to survey the environment since the larger population remains protected in biofilms (Fig. 3A).

## THE BIOFILM-DDR AXIS IN *A. BAUMANNII*

A factor that affects full eradication of *A. baumannii* infections is their ability to form biofilms, as indicated above, since they allow bacteria to resist antibiotic or environmental challenges such as desiccation (139–142). *A. baumannii* biofilms can adhere to both biotic and abiotic surfaces, including catheters and ventilators (143, 144), which is among the most common and with the highest mortality rates among *A. baumannii* infections (18, 145). While previous studies in *A. baumannii* have identified key genes involved in biofilm formation (see above), the mechanisms underlying the biofilm life cycle are still not fully understood.

An operon with a clear function in biofilms is those encoding adhesins (68, 146, 147). For most bacteria, the regulation of expression of these is well understood (148, 149), but not for *A. baumannii*. We will discuss here cell-surface attachment in *A. baumannii,* particularly to abiotic surfaces mediated by the Csu pili (74, 76, 78, 80). This pilus is one of the best understood in *A. baumannii,* including exquisite details about its structure (75). Due to this, Csu is an excellent marker of biofilm formation, since cell attachment is the first step required for it (68). The Csu pili is encoded in a single operon, consisting of the genes *csuAB*, *A*, *B*, *C*, *D*, and *E* (57). The pilus consists of CsuAB, CsuA, CsuB, and CsuE, with CsuAB being the major pilus subunit and CsuE being the tip subunit (57, 76, 78, 80). CsuC is involved in the trafficking of pilus components to CsuD, which facilitates polymerization of the pilus (76, 78, 79). Expression of the *csu* operon requires BfmR/S (59) (57), though BfmR does not bind to the *csu* promoter (63). VanR binds to the *csu* promoter and represses Csu operon expression (121). Induction occurs when vanillate binds to VanR, sequestering it from the promoter (121). Currently, the link between VanR and BfmR is unknown (Fig. 1). The possible mechanistic explanation for the VanR-vanillate pair’s role in activating *A. baumannii* attachment is unclear, though there is evidence indicating it has clinical relevance. A comparative transcriptome of *A. baumannii* strains resistant to antibiotics, including carbapenem, showed undetectable VanR expression when compared to sensitive strains (150), suggesting this is relevant in infection. This was the only gene downregulated in all antibiotic-resistant strains, regardless of the resistance marker (150). Examination of the Csu genes’ expression in the expression table provided by the authors showed consistently elevated levels of CsuA/B, suggesting the pili might be expressed. Unfortunately, expression analysis of other relevant genes was inconclusive, owing to high variability of the comparison group of sensitive strains (150).

Work in our lab established a link between biofilm development and the DDR in *A. baumannii* (Fig. 2 and 3). This link has been established in other bacteria, where biofilms and the DDR have either a direct (151) or an indirect correlation (138). In *E. coli* and *P. aeruginosa*, elevated DDR activity correlates with increased biofilm formation (152–155), whereas in *B. subtilis*, as in *A. baumannii*, the DDR is anticorrelated with biofilm development (120). Remarkably, Gram stain does not predict the direction of the biofilm-DDR relationship, despite the deep evolutionary separation between Firmicutes and Proteobacteria (156, 157).

We propose that whether the biofilm-DDR relationship is direct or inverse may depend on how RecA levels are regulated. Intriguingly, DDR-induced RecA levels in both *A. baumannii* and *B. subtilis* are ~4- and ~5-fold, respectively (120, 158, 159), while in *E. coli* these are ~10-fold higher (160). Basal RecA levels in *A. baumannii* are slightly higher than those in *E. coli* (115). Certain strains of *P. aeruginosa* also show an ~5-fold RecA increase in response to DNA damage, a higher basal level of RecA, and, like *A. baumannii*, exhibit an anticorrelation between biofilm formation and the DDR (161). For most other bacteria, RecA quantification is unknown, making any biofilm-DDR relationship unclear. As seen in other conserved pathways, RecA has a defined core activity in homologous recombination and the DDR, but the mechanistic details through which it carries out these processes may vary across species (162–164). RecA levels are likely a contributing factor, but other gene products probably also influence the direction of the biofilm-DDR relationship (Fig. 3).

## EPPR REPRESSES *RECA* AND REGULATES BIOFILM FORMATION AND DISPERSAL

∆*eppR* strains form virtually no biofilms, while high levels of EppR in a WT strain with a plasmid-borne copy of *eppR* under its own promoter phenocopy the WT biofilm (165). Given that EppR represses *recA* expression by binding directly to the DNA sequence transcribed into the 5′UTR of *recA* mRNA, and that RNA-seq data on the strain lacking EppR show a significant 2.5-fold increase in DdaA expression (165), it is possible that EppR may also be a DdaA repressor. Furthermore, there is evidence suggesting that EppR forms a protein complex with RecA (165).

**Fig 4:**
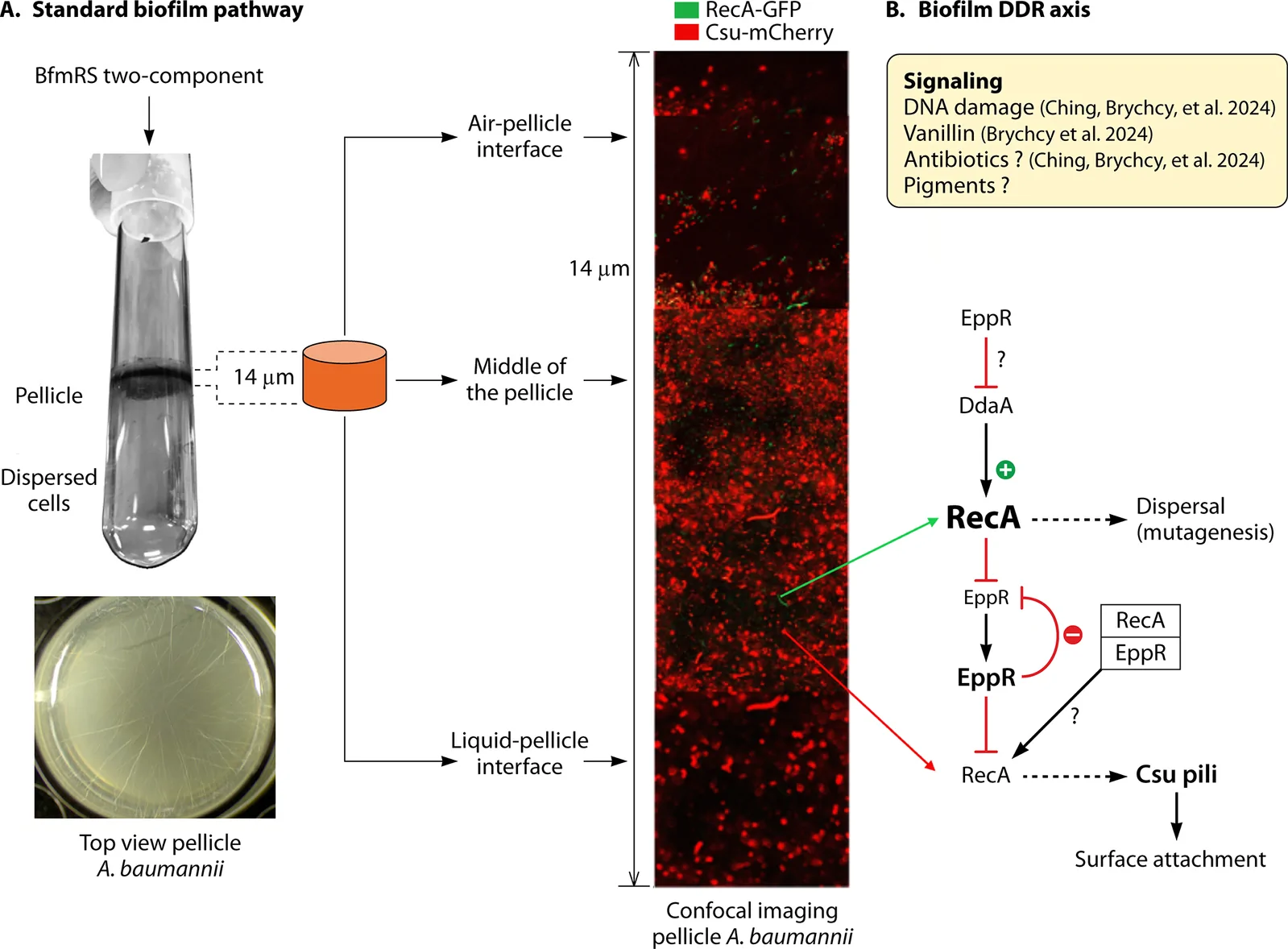
The biofilm DDR axis depends on at least RecA and EppR proteins. (**A**) Standard biofilm formation pathway. This is dependent on the two-component BfmRS system (33, 34). There is a likely overlap between panels **A** and **B**, as a large fraction of cells in stationary phase would have low RecA and not necessarily be in biofilms. However, these cells might be primed to form biofilms, and a small fraction are RecA high, which would not. (**B**) Biofilm-DDR axis. The confocal microscopy image is from an untreated WT *A. baumannii* ATCC 17978 strain pellicle biofilms formed in wells, as shown on the bottom left-hand side. The pellicle is ~14 µm in width. The cells carry an mCherry reporter of Csu pili expression and a GFP reporter of RecA. There are fewer cells that are GFP than those labeled mCherry. The opposite is true when the biofilms are treated with sublethal concentrations of ciprofloxacin (120). The model shown on the right-hand side of the figure displays the possible interplay of RecA and EppR. See text for more details.

Our current model of EppR function in biofilms is shown in Fig. 4. Confocal microscopy of biofilms made by cells expressing both RecA-GFP and *csu*-mCherry reporters shows that few cells within the biofilm have high RecA. These are present at the air-pellicle interface. These cells will likely experience more oxygen than the ones at the pellicle-liquid interface (Fig. 4). Under normal conditions with low DNA damage, there will be low levels of RecA, as DdaA would not be induced by ssDNA (Fig. 4, small font size RecA). Moreover, EppR levels would be high enough to repress *recA*. Under these conditions, *bfmR* expression would be activated by full-length UmuDAb (120), which would result in the induction of Csu pili and attachment. However, endogenous DNA damage within the biofilm or the addition of DNA-damaging agents, e.g., ciprofloxacin (120), would induce *ddaA* and *recA*, elevating its levels and resulting in a RecA-High subpopulation (Fig. 4, larger font RecA). These cells disperse (120).

In response to DNA damage, a fraction of cells would accumulate an as-yet-unidentified ligand that binds the sensor domain of EppR, sequestering it from its target promoters, including the *eppR* promoter itself (165) (Fig. 1 and 4). RecA would be elevated (Fig. 3B) in these cells because of the lack of EppR repression and *recA* activation by DdaA. In other cells, EppR levels would be maintained by autoregulation, and since RecA and EppR form a protein complex (165), RecA levels would be maintained low in biofilm in these cells, resulting in biofilm maintenance. This may be possible because RecA intracellular concentrations in *A. baumannii*, while similar to those of *E. coli* at baseline, are lower following DNA damage (120). Perhaps the RecA-EppR protein complex is part of a mechanism to maintain the biofilm cycle as highly dynamic. *A. baumannii* cells would be constantly dispersing from and returning to the biofilm pellicle or making new biofilms on available surfaces, which would in turn be highly dynamic. This also would explain why in *A. baumannii* there are clearly visible planktonic cells beneath the pellicle (120). This is not observed in, for example, *B. subtilis* or in environmental isolates from extreme environments (166). In these cases, the biofilms are clearly defined (166, 167), and there are no observable planktonic cells, i.e., these are very low. There seems to be a clearer distinction between cells in biofilms versus those that are not. We believe that in *A. baumannii,* there may be a “fuzzy” cell fate choice that would depend on the levels of DDR proteins, as well as likely others that may be biofilm-specific and DDR-independent (Fig. 4).

## MOVING FORWARD TO UNDERSTAND CELL FATE IN *A. BAUMANNII*

Here, we present an *A. baumannii* pathway to uncover cell fate decisions to stay or leave biofilms. There are many aspects that remain unclear, as pointed out throughout. It will be important to learn about the RecA, EppR, and DdaA protein thresholds needed to observe either biofilm formation or dispersal. Confocal microscopy could be used (120) to measure changes in the biofilm extracellular matrix as a marker of dispersal. Changes in the extracellular matrix are key since “dispersal” for *A. baumannii* may be different from that of flagellated bacteria that can literally swim their way out of the biofilm (68). *A. baumannii* does not possess flagella, so it must be able to somehow unstick from surfaces, perhaps coating itself with a highly hydrophilic surface that will allow it to slide (?) in a similar way to swarming (168). For example, Bap (86) would need to be downregulated in dispersing cells while type IV pili are upregulated (63). Fortunately, these are testable questions.

While the signal detected by the two-component sensor kinase BfmS remains elusive, it is clear that it is not needed to establish biofilms, as a deletion in *bfmS* results in phosphorylated BfmR leading to robust biofilms (63). There are alternative ways to phosphorylate BfmR independent of BfmS. Thus, it is likely there are distinct and perhaps overlapping mechanisms to establish and disperse biofilms in *A. baumannii*. The coming years will bring exciting results that hopefully will inform clinicians of the best ways to effectively contend with *A. baumannii* infections.

## References

[1] Biswas I, Rather PN, eds. 2019. Acinetobacter baumannii: Methods and Protocols (Methods in Molecular Biology). Vol. 1946.

[2] Yu Y, Mahdi R, Al-Hilfy Leon A, Vo N, Lofgren R, Mutabazi JL, Piepenbrink KH. 2025. Variation in type IV pilus stability modulates DNA uptake and biofilm formation. J Biol Chem 301:110787. doi:10.1016/j.jbc.2025.110787 41062070 PMC12603735

[3] Gopikrishnan M, Doss GPC. 2025. Targeting PilA in *Acinetobacter baumannii*: a computational approach for anti-virulent compound discovery. Mol Biotechnol 67:3906–3921. doi:10.1007/s12033-024-01300-9 39414707

[4] Jeong GJ, Khan F, Tabassum N, Kim YM. 2024. Motility of *Acinetobacter baumannii*: regulatory systems and controlling strategies. Appl Microbiol Biotechnol 108:3. doi:10.1007/s00253-023-12975-6 38159120 PMC13496788

[5] Kandolo O, Cherrak Y, Filella-Merce I, Le Guenno H, Kosta A, Espinosa L, Santucci P, Verthuy C, Lebrun R, Nilges M, Pellarin R, Durand E. 2023. *Acinetobacter* type VI secretion system comprises a non-canonical membrane complex. PLoS Pathog 19:e1011687. doi:10.1371/journal.ppat.1011687 37769028 PMC10564176

[6] Baumann P, Doudoroff M, Stanier RY. 1968. A study of the *Moraxella* group. II. Oxidative-negative species (genus *Acinetobacter*). J Bacteriol 95:1520–1541. doi:10.1128/jb.95.5.1520-1541.1968 5650064 PMC252171

[7] Towner KJ. 1992. The genus *Acinetobacter*, p 3137–3143. *In* Balows A, Trüper HG, Dworkin M, Harder W, Schleifer KH (ed), The prokaryotes: a handbook on the biology of bacteria: ecophysiology, isolation, identification, applications. Springer New York, New York, NY.

[8] Peleg AY, Seifert H, Paterson DL. 2008. *Acinetobacter baumannii*: emergence of a successful pathogen. Clin Microbiol Rev 21:538–582. doi:10.1128/CMR.00058-07 18625687 PMC2493088

[9] Karah N, Nemec A, Uhlin BE. 2025. History of the taxonomy of *Acinetobacter*: the emergence of hospital-adapted species of global health concern. Int J Syst Evol Microbiol 75:006983. doi:10.1099/ijsem.0.006983 41348474 PMC12680336

[10] Appaneal HJ, O’Neill E, Lopes VV, LaPlante KL, Caffrey AR. 2021. National trends in hospital, long-term care and outpatient *Acinetobacter baumannii* resistance rates. J Med Microbiol 70. doi:10.1099/jmm.0.001473 34919041

[11] Cerqueira GM, Peleg AY. 2011. Insights into *Acinetobacter baumannii* pathogenicity. IUBMB Life 63:1055–1060. doi:10.1002/iub.533 21989983

[12] Morris FC, Dexter C, Kostoulias X, Uddin MI, Peleg AY. 2019. The mechanisms of disease caused by *Acinetobacter baumannii*. Front Microbiol 10:1601. doi:10.3389/fmicb.2019.01601 31379771 PMC6650576

[13] Howard A, O’Donoghue M, Feeney A, Sleator RD. 2012. *Acinetobacter baumannii*: an emerging opportunistic pathogen. Virulence 3:243–250. doi:10.4161/viru.19700 22546906 PMC3442836

[14] Panosian Dunavan C. 2020. Superbug vs. superphage: a review of the perfect predator and an interview with Dr. Chip Schooley. Am J Trop Med Hyg 102:245–248. doi:10.4269/ajtmh.19-0770 31802734 PMC6947799

[15] Shaikh A, Marathe A, Prajapati B. 2025. Genomic analysis of virulence factors of nosocomial MDR *A. baumannii* from a tertiary care hospital. Microbiol Spectr 13:e0070725. doi:10.1128/spectrum.00707-25 41217201 PMC12671222

[16] Ballarin A, Posteraro B, Demartis G, Gervasi S, Panzarella F, Torelli R, Paroni Sterbini F, Morandotti G, Posteraro P, Ricciardi W, Gervasi Vidal KA, Sanguinetti M. 2014. Forecasting ESKAPE infections through a time-varying auto-adaptive algorithm using laboratory-based surveillance data. BMC Infect Dis 14:634. doi:10.1186/s12879-014-0634-9 25480675 PMC4266976

[17] Boucher HW, Talbot GH, Bradley JS, Edwards JE, Gilbert D, Rice LB, Scheld M, Spellberg B, Bartlett J. 2009. Bad bugs, no drugs: no ESKAPE! An update from the Infectious Diseases Society of America. Clin Infect Dis 48:1–12. doi:10.1086/595011 19035777

[18] Antunes LCS, Visca P, Towner KJ. 2014. *Acinetobacter baumannii*: evolution of a global pathogen. Pathog Dis 71:292–301. doi:10.1111/2049-632X.12125 24376225

[19] Bergogne-Bérézin E, Towner KJ. 1996. *Acinetobacter* spp. as nosocomial pathogens: microbiological, clinical, and epidemiological features. Clin Microbiol Rev 9:148–165. doi:10.1128/CMR.9.2.148 8964033 PMC172888

[20] McConnell MJ, Actis L, Pachón J. 2013. *Acinetobacter baumannii*: human infections, factors contributing to pathogenesis and animal models. FEMS Microbiol Rev 37:130–155. doi:10.1111/j.1574-6976.2012.00344.x 22568581

[21] Roca I, Espinal P, Vila-Farrés X, Vila J. 2012. The *Acinetobacter baumannii* oxymoron: commensal hospital dweller turned pan-drug-resistant menace. Front Microbiol 3:148. doi:10.3389/fmicb.2012.00148 22536199 PMC3333477

[22] Makwana N, Karad DD Dheeraj Yadav B, Saini C, Mercier C, Kharat AS. 2026. Global strategies to fight carbapenem-resistant *Acinetobacter baumannii* (CRAB) infections. Microb Pathog 214:108412. doi:10.1016/j.micpath.2026.108412 41771383

[23] Anand G, Lahariya R, Priyadarshi K. 2026. The rise of WHO-priority pathogens in central line associated bloodstream infection: challenging the AWaRe paradigm in critical care. World J Microbiol Biotechnol 42:55. doi:10.1007/s11274-026-04797-1 41571937

[24] Vicentini C, Bussolino R, Kuczewski E, Elias C, Machut A, Bettoni S, Perego M, Bresciano L, Ramondetti L, Friggeri A, Lepape A, Zotti CM, Finazzi S, Collaborating group. 2026. Carbapenem-resistant *Acinetobacter baumannii* infections in intensive care units: incidence, infection prevention and control, and antimicrobial stewardship practices in the cross-border region between Italy and France, 2019–2022. J Hosp Infect 170:174–183. doi:10.1016/j.jhin.2026.01.019 41655744

[25] Chang WN, Lu CH, Huang CR, Chuang YC. 2000. Community-acquired *Acinetobacter meningitis* in adults. Infection 28:395–397. doi:10.1007/s150100070013 11139162

[26] Falagas ME, Karveli EA, Kelesidis I, Kelesidis T. 2007. Community-acquired *Acinetobacter* infections. Eur J Clin Microbiol Infect Dis 26:857–868. doi:10.1007/s10096-007-0365-6 17701432

[27] Falagas ME, Rafailidis PI. 2007. Attributable mortality of *Acinetobacter baumannii*: no longer a controversial issue. Crit Care 11:134. doi:10.1186/cc5911 17543135 PMC2206403

[28] Dexter C, Murray GL, Paulsen IT, Peleg AY. 2015. Community-acquired *Acinetobacter baumannii*: clinical characteristics, epidemiology and pathogenesis. Expert Rev Anti Infect Ther 13:567–573. doi:10.1586/14787210.2015.1025055 25850806

[29] Serota DP, Sexton ME, Kraft CS, Palacio F. 2018. Severe community-acquired pneumonia due to *Acinetobacter baumannii* in North America: case report and review of the literature. Open Forum Infect Dis 5:fy044. doi:10.1093/ofid/ofy044 PMC5846288 29564365

[30] Leung WS, Chu CM, Tsang KY, Lo FH, Lo KF, Ho PL. 2006. Fulminant community-acquired *Acinetobacter baumannii* pneumonia as a distinct clinical syndrome. Chest 129:102–109. doi:10.1378/chest.129.1.102 16424419

[31] Green ER, Fakhoury JN, Monteith AJ, Pi H, Giedroc DP, Skaar EP. 2022. Bacterial hydrophilins promote pathogen desiccation tolerance. Cell Host Microbe 30:975–987. doi:10.1016/j.chom.2022.03.019 35413266 PMC9283220

[32] Weber BS, Kinsella RL, Harding CM, Feldman MF. 2017. The secrets of *Acinetobacter* secretion. Trends Microbiol 25:532–545. doi:10.1016/j.tim.2017.01.005 28216293 PMC5474180

[33] Weber BS, Ly PM, Irwin JN, Pukatzki S, Feldman MF. 2015. A multidrug resistance plasmid contains the molecular switch for type VI secretion in *Acinetobacter baumannii*. Proc Natl Acad Sci U S A 112:9442–9447. doi:10.1073/pnas.1502966112 26170289 PMC4522760

[34] Gordon NC, Wareham DW. 2010. Multidrug-resistant *Acinetobacter baumannii*: mechanisms of virulence and resistance. Int J Antimicrob Agents 35:219–226. doi:10.1016/j.ijantimicag.2009.10.024 20047818

[35] Taccone FS, Rodriguez-Villalobos H, De Backer D, De Moor V, Deviere J, Vincent J-L, Jacobs F. 2006. Successful treatment of septic shock due to pan-resistant *Acinetobacter baumannii* using combined antimicrobial therapy including tigecycline. Eur J Clin Microbiol Infect Dis 25:257–260. doi:10.1007/s10096-006-0123-1 16572310

[36] CDC. 2019. Antibiotic Resistance Threats in the United States, 2019. U.S. Department of Health and Human Services, CDC, Atlanta, GA. Available from: www.cdc.gov/DrugResistance/Biggest-Threats.html

[37] Banerjee T, Mishra K, Rakshit P, Sharma S, Garg R, Das P, Bhattacharjee A, Kumar S, Kumar R, Anupurba S. 2026. Susceptibility of carbapenem-resistant *Acinetobacter baumannii* and carbapenem-resistant *Klebsiella pneumoniae* against cefiderocol with reference to their genetic profile in India. Microb Drug Resist 32:111–118. doi:10.1177/10766294261426703 41783919

[38] Chukamnerd A, Surachat K, Pomwised R, Palittapongarnpim P, Singkhamanan K, Chusri S. 2026. Genomic epidemiology of carbapenem-resistant *Acinetobacter baumannii* isolated from patients admitted to intensive care units in network hospitals in Southern Thailand. Antibiotics (Basel) 15:133. doi:10.3390/antibiotics15020133 41750431 PMC12937475

[39] Lee HJ, Lee SJ, Sung YH, Park SM, Choi SP, Kim Y, Yoon YK. 2026. Peptide-based antimicrobial effect against carbapenem-resistant *Acinetobacter baumannii*: preclinical drug assessment and translational potential. Front Pharmacol 17:1732644. doi:10.3389/fphar.2026.1732644 41788804 PMC12957892

[40] Costerton JW. 1999. Introduction to biofilm. Int J Antimicrob Agents 11:217–221; doi:10.1016/s0924-8579(99)00018-7 10394973

[41] Costerton JW, Cheng KJ, Geesey GG, Ladd TI, Nickel JC, Dasgupta M, Marrie TJ. 1987. Bacterial biofilms in nature and disease. Annu Rev Microbiol 41:435–464. doi:10.1146/annurev.mi.41.100187.002251 3318676

[42] Costerton JW, Montanaro L, Arciola CR. 2005. Biofilm in implant infections: its production and regulation. Int J Artif Organs 28:1062–1068. doi:10.1177/039139880502801103 16353112

[43] Costerton JW, Stewart PS, Greenberg EP. 1999. Bacterial biofilms: a common cause of persistent infections. Science 284:1318–1322. doi:10.1126/science.284.5418.1318 10334980

[44] Dar D, Dar N, Cai L, Newman DK. 2021. Spatial transcriptomics of planktonic and sessile bacterial populations at single-cell resolution. Science 373:eabi4882. doi:10.1126/science.abi4882 34385369 PMC8454218

[45] Shakib NH, Hashemizadeh Z, Zomorodi AR, Khashei R, Sadeghi Y, Bazargani A. 2025. Evaluation of the relatedness between the biofilm-associated genes and antimicrobial resistance among *Acinetobacter baumannii* isolates in the southwest Iran. Iran J Microbiol 17:80–91. doi:10.18502/ijm.v17i1.17804 40330064 PMC12049743

[46] Wiradiputra MRD, Khuntayaporn P, Thirapanmethee K, Wanapaisan P, Chomnawang MT. 2025. Genomic insights into biofilm-associated virulence in extensively drug-resistant *Acinetobacter baumannii*. Curr Res Microb Sci 9:100434. doi:10.1016/j.crmicr.2025.100434 40686839 PMC12272471

[47] Choudhary J, Shariff M. 2025. Characterization of carbapenem-resistant biofilm forming *Acinetobacter baumannii* isolates from clinical and surveillance samples. Sci Rep 15:33892. doi:10.1038/s41598-025-09530-w 41028035 PMC12484597

[48] Foster KR. 2005. Biomedicine. Hamiltonian medicine: why the social lives of pathogens matter. Science 308:1269–1270. doi:10.1126/science.1108158 15919984

[49] Gristina AG, Oga M, Webb LX, Hobgood CD. 1985. Adherent bacterial colonization in the pathogenesis of osteomyelitis. Science 228:990–993. doi:10.1126/science.4001933 4001933

[50] Costerton JW, Lewandowski Z, Caldwell DE, Korber DR, Lappin-Scott HM. 1995. Microbial biofilms. Annu Rev Microbiol 49:711–745. doi:10.1146/annurev.mi.49.100195.003431 8561477

[51] Wang S, Zhao Y, Breslawec AP, Liang T, Deng Z, Kuperman LL, Yu Q. 2023. Strategy to combat biofilms: a focus on biofilm dispersal enzymes. NPJ Biofilms Microbiomes 9:63. doi:10.1038/s41522-023-00427-y 37679355 PMC10485009

[52] Wille J, Coenye T. 2020. Biofilm dispersion: the key to biofilm eradication or opening Pandora’s box? Biofilm 2:100027. doi:10.1016/j.bioflm.2020.100027 33447812 PMC7798462

[53] Prentice JA, Kasivisweswaran S, van de Weerd R, Bridges AA. 2024. Biofilm dispersal patterns revealed using far-red fluorogenic probes. PLoS Biol 22:e3002928. doi:10.1371/journal.pbio.3002928 39585926 PMC11627390

[54] Johnson GE, Fei C, Wingreen NS, Bassler BL. 2025. Analysis of gene expression within individual cells reveals spatiotemporal patterns underlying *Vibrio cholerae* biofilm development. PLoS Biol 23:e3003187. doi:10.1371/journal.pbio.3003187 40378130 PMC12121927

[55] Clemmer KM, Bonomo RA, Rather PN. 2011. Genetic analysis of surface motility in *Acinetobacter baumannii*. Microbiology 157:2534–2544. doi:10.1099/mic.0.049791-0 21700662 PMC3352170

[56] Ronish LA, Lillehoj E, Fields JK, Sundberg EJ, Piepenbrink KH. 2019. The structure of PilA from *Acinetobacter baumannii* AB5075 suggests a mechanism for functional specialization in *Acinetobacter* type IV pili. J Biol Chem 294:218–230. doi:10.1074/jbc.RA118.005814 30413536 PMC6322890

[57] Tomaras AP, Flagler MJ, Dorsey CW, Gaddy JA, Actis LA. 2008. Characterization of a two-component regulatory system from *Acinetobacter baumannii* that controls biofilm formation and cellular morphology. Microbiology (Reading) 154:3398–3409. doi:10.1099/mic.0.2008/019471-0 18957593

[58] Draughn GL, Milton ME, Feldmann EA, Bobay BG, Roth BM, Olson AL, Thompson RJ, Actis LA, Davies C, Cavanagh J. 2018. The structure of the biofilm-controlling response regulator BfmR from *Acinetobacter baumannii* reveals details of its DNA-binding mechanism. J Mol Biol 430:806–821. doi:10.1016/j.jmb.2018.02.002 29438671 PMC5861000

[59] Gaddy JA, Actis LA. 2009. Regulation of *Acinetobacter baumannii* biofilm formation. Future Microbiol 4:273–278. doi:10.2217/fmb.09.5 19327114 PMC2724675

[60] Geisinger E, Mortman NJ, Vargas-Cuebas G, Tai AK, Isberg RR. 2018. A global regulatory system links virulence and antibiotic resistance to envelope homeostasis in *Acinetobacter baumannii*. PLoS Pathog 14:e1007030. doi:10.1371/journal.ppat.1007030 29795704 PMC5967708

[61] Krasauskas R, Skerniškytė J, Armalytė J, Sužiedėlienė E. 2019. The role of *Acinetobacter baumannii* response regulator BfmR in pellicle formation and competitiveness via contact-dependent inhibition system. BMC Microbiol 19:241. doi:10.1186/s12866-019-1621-5 31690263 PMC6833216

[62] Palethorpe S, Farrow JM III, Wells G, Milton ME, Actis LA, Cavanagh J, Pesci EC. 2022. *Acinetobacter baumannii* regulates its stress responses via the BfmRS two-component regulatory system. J Bacteriol 204:e0049421. doi:10.1128/jb.00494-21 34871031 PMC8846317

[63] Raustad N, Dai Y, Iinishi A, Mohapatra A, Soo MW, Hay E, Hernandez GM, Geisinger E. 2025. A phosphorylation signal activates genome-wide transcriptional control by BfmR, the global regulator of *Acinetobacter* resistance and virulence. Nucleic Acids Res 53:gkaf063. doi:10.1093/nar/gkaf063 39921563 PMC11806355

[64] Upmanyu K, Haq QMR, Singh R. 2022. Factors mediating *Acinetobacter baumannii* biofilm formation: opportunities for developing therapeutics. Curr Res Microb Sci 3:100131. doi:10.1016/j.crmicr.2022.100131 35909621 PMC9325880

[65] Gaddy JA, Tomaras AP, Actis LA. 2009. The *Acinetobacter baumannii* 19606 OmpA protein plays a role in biofilm formation on abiotic surfaces and in the interaction of this pathogen with eukaryotic cells. Infect Immun 77:3150–3160. doi:10.1128/IAI.00096-09 19470746 PMC2715673

[66] Bai J, Raustad N, Denoncourt J, van Opijnen T, Geisinger E. 2023. Genome-wide phage susceptibility analysis in *Acinetobacter baumannii* reveals capsule modulation strategies that determine phage infectivity. PLoS Pathog 19:e1010928. doi:10.1371/journal.ppat.1010928 37289824 PMC10249906

[67] Sadiq FA, Flint S, Li Y, Liu T, Lei Y, Sakandar HA, He G. 2017. New mechanistic insights into the motile-to-sessile switch in various bacteria with particular emphasis on *Bacillus subtilis* and *Pseudomonas aeruginosa*: a review. Biofouling 33:306–326. doi:10.1080/08927014.2017.1304541 28347177

[68] Vlamakis H, Chai Y, Beauregard P, Losick R, Kolter R. 2013. Sticking together: building a biofilm the *Bacillus subtilis* way. Nat Rev Microbiol 11:157–168. doi:10.1038/nrmicro2960 23353768 PMC3936787

[69] Geisinger E, Isberg RR. 2015. Antibiotic modulation of capsular exopolysaccharide and virulence in *Acinetobacter baumannii*. PLoS Pathog 11:e1004691. doi:10.1371/journal.ppat.1004691 25679516 PMC4334535

[70] de Breij A, Gaddy J, van der Meer J, Koning R, Koster A, van den Broek P, Actis L, Nibbering P, Dijkshoorn L. 2009. CsuA/BABCDE-dependent pili are not involved in the adherence of *Acinetobacter baumannii* ATCC19606^T^ to human airway epithelial cells and their inflammatory response. Res Microbiol 160:213–218. doi:10.1016/j.resmic.2009.01.002 19530313

[71] Vila-Farrés X, Parra-Millán R, Sánchez-Encinales V, Varese M, Ayerbe-Algaba R, Bayó N, Guardiola S, Pachón-Ibáñez ME, Kotev M, García J, Teixidó M, Vila J, Pachón J, Giralt E, Smani Y. 2017. Combating virulence of Gram-negative bacilli by OmpA inhibition. Sci Rep 7:14683. doi:10.1038/s41598-017-14972-y 29089624 PMC5666006

[72] Yaeger LN, Ranieri MRM, Chee J, Karabelas-Pittman S, Rudolph M, Giovannoni AM, Harvey H, Burrows LL. 2024. A genetic screen identifies a role for *oprF* in *Pseudomonas aeruginosa* biofilm stimulation by subinhibitory antibiotics. NPJ Biofilms Microbiomes 10:30. doi:10.1038/s41522-024-00496-7 38521769 PMC10960818

[73] Zhao D, Zeng TY, Wang B, Li W, Zhao DG, Zhang XY. 2026. Effect of OmpA of *Acinetobacter baumannii* on apoptosis in pulmonary epithelial cells and its molecular mechanism. Microb Pathog 210:108193. doi:10.1016/j.micpath.2025.108193 41260154

[74] Ahmad I, Nadeem A, Mushtaq F, Zlatkov N, Shahzad M, Zavialov AV, Wai SN, Uhlin BE. 2023. Csu pili dependent biofilm formation and virulence of *Acinetobacter baumannii*. NPJ Biofilms Microbiomes 9:101. doi:10.1038/s41522-023-00465-6 38097635 PMC10721868

[75] Malmi H, Pakharukova N, Paul B, Tuittila M, Ahmad I, Knight SD, Uhlin BE, Ghosal D, Zavialov AV. 2026. Antiparallel stacking of Csu pili drives *Acinetobacter baumannii* 3D biofilm assembly. Nat Commun 17:2508. doi:10.1038/s41467-026-68860-z 41654547 PMC12996565

[76] Pakharukova N, Garnett JA, Tuittila M, Paavilainen S, Diallo M, Xu Y, Matthews SJ, Zavialov AV. 2015. Structural insight into archaic and alternative chaperone-usher pathways reveals a novel mechanism of pilus biogenesis. PLoS Pathog 11:e1005269. doi:10.1371/journal.ppat.1005269 26587649 PMC4654587

[77] Pakharukova N, Malmi H, Tuittila M, Dahlberg T, Ghosal D, Chang Y-W, Myint SL, Paavilainen S, Knight SD, Lamminmäki U, Uhlin BE, Andersson M, Jensen G, Zavialov AV. 2022. Archaic chaperone-usher pili self-secrete into superelastic zigzag springs. Nature 609:335–340. doi:10.1038/s41586-022-05095-0 35853476 PMC9452303

[78] Pakharukova N, McKenna S, Tuittila M, Paavilainen S, Malmi H, Xu Y, Parilova O, Matthews S, Zavialov AV. 2018. Archaic and alternative chaperones preserve pilin folding energy by providing incomplete structural information. J Biol Chem 293:17070–17080. doi:10.1074/jbc.RA118.004170 30228191 PMC6222105

[79] Pakharukova N, Tuittila M, Paavilainen S, Malmi H, Parilova O, Teneberg S, Knight SD, Zavialov AV. 2018. Structural basis for *Acinetobacter baumannii* biofilm formation. Proc Natl Acad Sci USA 115:5558–5563. doi:10.1073/pnas.1800961115 29735695 PMC6003481

[80] Pakharukova N, Tuittila M, Paavilainen S, Zavialov A. 2015. Crystallization and preliminary X-ray diffraction analysis of the Csu pili CsuC-CsuA/B chaperone-major subunit pre-assembly complex from *Acinetobacter baumannii*. Acta Crystallogr F Struct Biol Commun 71:770–774. doi:10.1107/S2053230X15007955 26057810 PMC4461345

[81] Pakharukova N, Tuittila M, Paavilainen S, Zavialov A. 2017. Methylation, crystallization and SAD phasing of the Csu pilus CsuC-CsuE chaperone-adhesin subunit pre-assembly complex from *Acinetobacter baumannii*. Acta Crystallogr F Struct Biol Commun 73:450–454. doi:10.1107/S2053230X17009566 28777087 PMC5544001

[82] Harding CM, Pulido MR, Di Venanzio G, Kinsella RL, Webb AI, Scott NE, Pachón J, Feldman MF. 2017. Pathogenic *Acinetobacter* species have a functional type I secretion system and contact-dependent inhibition systems. J Biol Chem 292:9075–9087. doi:10.1074/jbc.M117.781575 28373284 PMC5454093

[83] Loehfelm TW, Luke NR, Campagnari AA. 2008. Identification and characterization of an *Acinetobacter baumannii* biofilm-associated protein. J Bacteriol 190:1036–1044. doi:10.1128/JB.01416-07 18024522 PMC2223572

[84] Taglialegna A, Navarro S, Ventura S, Garnett JA, Matthews S, Penades JR, Lasa I, Valle J. 2016. Staphylococcal bap proteins build amyloid scaffold biofilm matrices in response to environmental signals. PLoS Pathog 12:e1005711. doi:10.1371/journal.ppat.1005711 27327765 PMC4915627

[85] Valle J, Fang X, Lasa I. 2020. Revisiting bap multidomain protein: more than sticking bacteria together. Front Microbiol 11:613581. doi:10.3389/fmicb.2020.613581 33424817 PMC7785521

[86] Goh HMS, Beatson SA, Totsika M, Moriel DG, Phan M-D, Szubert J, Runnegar N, Sidjabat HE, Paterson DL, Nimmo GR, Lipman J, Schembri MA. 2013. Molecular analysis of the *Acinetobacter baumannii* biofilm-associated protein. Appl Environ Microbiol 79:6535–6543. doi:10.1128/AEM.01402-13 23956398 PMC3811493

[87] Böhning J, Ghrayeb M, Pedebos C, Abbas DK, Khalid S, Chai L, Bharat TAM. 2022. Donor-strand exchange drives assembly of the TasA scaffold in *Bacillus subtilis* biofilms. Nat Commun 13:7082. doi:10.1038/s41467-022-34700-z 36400765 PMC9674648

[88] Cámara-Almirón J, Navarro Y, Díaz-Martínez L, Magno-Pérez-Bryan MC, Molina-Santiago C, Pearson JR, de Vicente A, Pérez-García A, Romero D. 2020. Dual functionality of the amyloid protein TasA in *Bacillus* physiology and fitness on the phylloplane. Nat Commun 11:1859. doi:10.1038/s41467-020-15758-z 32313019 PMC7171179

[89] Ayswarya K, Ibrahim R, Veetilvalappil VV, Bhat NB, Aranjani JM. 2026. Virulence arsenal of *Acinetobacter baumannii*: mechanisms driving persistence and resistance. Arch Microbiol 208:162. doi:10.1007/s00203-025-04668-7 41627435 PMC12864274

[90] Brossard KA, Campagnari AA. 2012. The *Acinetobacter baumannii* biofilm-associated protein plays a role in adherence to human epithelial cells. Infect Immun 80:228–233. doi:10.1128/IAI.05913-11 22083703 PMC3255684

[91] Satchell KJF. 2011. Structure and function of MARTX toxins and other large repetitive RTX proteins. Annu Rev Microbiol 65:71–90. doi:10.1146/annurev-micro-090110-102943 21639783

[92] Coyne S, Courvalin P, Périchon B. 2011. Efflux-mediated antibiotic resistance in *Acinetobacter* spp. Antimicrob Agents Chemother 55:947–953. doi:10.1128/AAC.01388-10 21173183 PMC3067115

[93] Coyne S, Rosenfeld N, Lambert T, Courvalin P, Périchon B. 2010. Overexpression of resistance-nodulation-cell division pump AdeFGH confers multidrug resistance in *Acinetobacter baumannii*. Antimicrob Agents Chemother 54:4389–4393. doi:10.1128/AAC.00155-10 20696879 PMC2944555

[94] He X, Lu F, Yuan F, Jiang D, Zhao P, Zhu J, Cheng H, Cao J, Lu G. 2015. Biofilm formation caused by clinical *Acinetobacter baumannii* isolates is associated with overexpression of the AdeFGH Efflux Pump. Antimicrob Agents Chemother 59:4817–4825. doi:10.1128/AAC.00877-15 26033730 PMC4505227

[95] Ching C, Yang B, Onwubueke C, Lazinski D, Camilli A, Godoy VG. 2019. Lon protease has multifaceted biological functions in *Acinetobacter baumannii*. J Bacteriol 201:e00536-18. doi:10.1128/JB.00536-18 30348832 PMC6304660

[96] Nait Chabane Y, Marti S, Rihouey C, Alexandre S, Hardouin J, Lesouhaitier O, Vila J, Kaplan JB, Jouenne T, Dé E. 2014. Characterisation of pellicles formed by *Acinetobacter baumannii* at the Air-liquid interface. PLoS One 9:e111660. doi:10.1371/journal.pone.0111660 25360550 PMC4216135

[97] Sahu PK, Iyer PS, Oak AM, Pardesi KR, Chopade BA. 2012. Characterization of eDNA from the clinical strain *Acinetobacter baumannii* AIIMS 7 and its role in biofilm formation. ScientificWorldJournal 2012:973436. doi:10.1100/2012/973436 22593716 PMC3346689

[98] Aranda J, Bardina C, Beceiro A, Rumbo S, Cabral MP, Barbé J, Bou G. 2011. *Acinetobacter baumannii* RecA protein in repair of DNA damage, antimicrobial resistance, general stress response, and virulence. J Bacteriol 193:3740–3747. doi:10.1128/JB.00389-11 21642465 PMC3147500

[99] Norton MD, Spilkia AJ, Godoy VG. 2013. Antibiotic resistance acquired through a DNA damage-inducible response in *Acinetobacter baumannii*. J Bacteriol 195:1335–1345. doi:10.1128/JB.02176-12 23316046 PMC3591989

[100] Radman M. 1975. SOS repair hypothesis: phenomenology of an inducible DNA repair which is accompanied by mutagenesis. Basic Life Sci 5A:355–367. doi:10.1007/978-1-4684-2895-7_48 1103845

[101] Witkin EM. 1967. The radiation sensitivity of *Escherichia coli* B: a hypothesis relating filament formation and prophage induction. Proc Natl Acad Sci USA 57:1275–1279. doi:10.1073/pnas.57.5.1275 5341236 PMC224468

[102] Simmons LA, Foti JJ, Cohen SE, Walker GC. 2008. The SOS regulatory network. EcoSal Plus 2008. doi:10.1128/ecosalplus.5.4.3 PMC4196698 25325076

[103] Sutton MD, Kim M, Walker GC. 2001. Genetic and biochemical characterization of a novel *umuD* mutation: insights into a mechanism for UmuD self-cleavage. J Bacteriol 183:347–357. doi:10.1128/JB.183.1.347-357.2001 11114935 PMC94884

[104] Janion C. 2008. Inducible SOS response system of DNA repair and mutagenesis in *Escherichia coli*. Int J Biol Sci 4:338–344. doi:10.7150/ijbs.4.338 18825275 PMC2556049

[105] Little JW, Mount DW. 1982. The SOS regulatory system of *Escherichia coli*. Cell 29:11–22. doi:10.1016/0092-8674(82)90085-X 7049397

[106] Maslowska KH, Makiela‐Dzbenska K, Fijalkowska IJ. 2019. The SOS system: a complex and tightly regulated response to DNA damage. Environ Mol Mutagen 60:368–384. doi:10.1002/em.22267 30447030 PMC6590174

[107] Walker GC. 1984. Mutagenesis and inducible responses to deoxyribonucleic acid damage in *Escherichia coli*. Microbiol Rev 48:60–93. doi:10.1128/mr.48.1.60-93.1984 6371470 PMC373003

[108] Godoy VG, Beuning PJ, Walker GC. 2005. Gene expression in bacterial systems: the LexA regulatory system. *In* Encyclopedia of biological chemistry. Academic Press/Elsevier Science, San Diego, CA.

[109] Courcelle J, Khodursky A, Peter B, Brown PO, Hanawalt PC. 2001. Comparative gene expression profiles following UV exposure in wild-type and SOS-deficient *Escherichia coli*. Genetics 158:41–64. doi:10.1093/genetics/158.1.41 11333217 PMC1461638

[110] Maso L, Vascon F, Chinellato M, Goormaghtigh F, Bellio P, Campagnaro E, Van Melderen L, Ruzzene M, Pardon E, Angelini A, Celenza G, Steyaert J, Tondi D, Cendron L. 2022. Nanobodies targeting LexA autocleavage disclose a novel suppression strategy of SOS-response pathway. Structure 30:1479–1493. doi:10.1016/j.str.2022.09.004 36240773

[111] Jones EC, Uphoff S. 2021. Single-molecule imaging of LexA degradation in *Escherichia coli* elucidates regulatory mechanisms and heterogeneity of the SOS response. Nat Microbiol 6:981–990. doi:10.1038/s41564-021-00930-y 34183814 PMC7611437

[112] Goodman MF, McDonald JP, Jaszczur MM, Woodgate R. 2016. Insights into the complex levels of regulation imposed on *Escherichia coli* DNA polymerase V. DNA Repair (Amst) 44:42–50. doi:10.1016/j.dnarep.2016.05.005 27236212 PMC4958523

[113] Cook D, Flannigan MD, Chariker JH, Hare JM. 2023. DNA damage response coregulator *ddrR* affects many cellular pathways and processes in *Acinetobacter baumannii* 17978. Front Cell Infect Microbiol 13:1324091. doi:10.3389/fcimb.2023.1324091 38274737 PMC10808703

[114] Robinson A, Brzoska AJ, Turner KM, Withers R, Harry EJ, Lewis PJ, Dixon NE. 2010. Essential biological processes of an emerging pathogen: DNA replication, transcription, and cell division in *Acinetobacter* spp. Microbiol Mol Biol Rev 74:273–297. doi:10.1128/MMBR.00048-09 20508250 PMC2884411

[115] Macguire AE, Ching MC, Diamond BH, Kazakov A, Novichkov P, Godoy VG. 2014. Activation of phenotypic subpopulations in response to ciprofloxacin treatment in *Acinetobacter baumannii*. Mol Microbiol 92:138–152. doi:10.1111/mmi.12541 24612352 PMC4005408

[116] Ahluwalia D, Schaaper RM. 2013. Hypermutability and error catastrophe due to defects in ribonucleotide reductase. Proc Natl Acad Sci USA 110:18596–18601. doi:10.1073/pnas.1310849110 24167285 PMC3832021

[117] Song S, Zhong S, Shi Q, Zheng X, Yao Y, Wang W, Chen S, Huang Z, An D, Xu H, Tian B, Zhao Y, Wang L, Zhang W, Hua X, Yu Y, Lu H, Fan L, Hua Y. 2025. An activator regulates the DNA damage response and anti-phage defense networks in *Moraxellaceae*. Nucleic Acids Res 53:gkaf828. doi:10.1093/nar/gkaf828 40874593 PMC12392091

[118] Ching C, Gozzi K, Heinemann B, Chai Y, Godoy VG. 2017. RNA-mediated *cis* regulation in *Acinetobacter baumannii* modulates stress-induced phenotypic variation. J Bacteriol 199:e00799-16. doi:10.1128/JB.00799-16 28320880 PMC5424255

[119] Hare JM, Ferrell JC, Witkowski TA, Grice AN. 2014. Prophage induction and differential RecA and UmuDAb transcriptome regulation in the DNA damage responses of *Acinetobacter baumannii* and *Acinetobacter baylyi*. PLoS One 9:e93861. doi:10.1371/journal.pone.0093861 24709747 PMC3978071

[120] Ching C, Brychcy M, Nguyen B, Muller P, Pearson AR, Downs M, Regan S, Isley B, Fowle W, Chai Y, Godoy VG. 2024. RecA levels modulate biofilm development in *Acinetobacter baumannii*. Mol Microbiol 121:196–212. doi:10.1111/mmi.15188 37918886

[121] Brychcy M, Nguyen B, Tierney GA, Casula P, Kokodynski A, Godoy VG. 2024. The metabolite vanillic acid regulates *Acinetobacter baumannii* surface attachment. Mol Microbiol 121:833–849. doi:10.1111/mmi.15234 38308563

[122] Witkowski TA, Grice AN, Stinnett DB, Wells WK, Peterson MA, Hare JM. 2016. UmuDAb: an error-prone polymerase accessory homolog whose N-terminal domain is required for repression of DNA damage inducible gene expression in *Acinetobacter baylyi*. PLoS One 11:e0152013. doi:10.1371/journal.pone.0152013 27010837 PMC4807011

[123] Hare JM, Perkins SN, Gregg-Jolly LA. 2006. A constitutively expressed, truncated *umuDC* operon regulates the *recA*-dependent DNA damage induction of a gene in *Acinetobacter baylyi* strain ADP1. Appl Environ Microbiol 72:4036–4043. doi:10.1128/AEM.02774-05 16751513 PMC1489636

[124] Lima-Noronha MA, Fonseca DLH, Oliveira RS, Freitas RR, Park JH, Galhardo RS. 2022. Sending out an SOS - the bacterial DNA damage response. Genet Mol Biol 45:e20220107. doi:10.1590/1678-4685-GMB-2022-0107 36288458 PMC9578287

[125] Hare JM, Adhikari S, Lambert KV, Hare AE, Grice AN. 2012. The *Acinetobacter* regulatory UmuDAb protein cleaves in response to DNA damage with chimeric LexA/UmuD characteristics. FEMS Microbiol Lett 334:57–65. doi:10.1111/j.1574-6968.2012.02618.x 22697494 PMC3475745

[126] Peterson MA, Grice AN, Hare JM. 2020. A corepressor participates in LexA-independent regulation of error-prone polymerases in *Acinetobacter*. Microbiology (Reading) 166:212–226. doi:10.1099/mic.0.000866 31687925 PMC7273328

[127] Candra B, Cook D, Hare J. 2024. Repression of *Acinetobacter baumannii* DNA damage response requires DdrR-assisted binding of UmuDAb dimers to atypical SOS box. J Bacteriol 206:e0043223. doi:10.1128/jb.00432-23 38727225 PMC11332147

[128] Cook D, Flannigan MD, Candra BV, Compton KD, Hare JM. 2022. The DdrR coregulator of the *Acinetobacter baumannii* mutagenic DNA damage response potentiates UmuDAb repression of error-prone polymerases. J Bacteriol 204:e0016522. doi:10.1128/jb.00165-22 36194009 PMC9664961

[129] Pavlin A, Bajc G, Fornelos N, Browning DF, Butala M. 2022. The small DdrR protein directly interacts with the UmuDAb regulator of the mutagenic DNA damage response in *Acinetobacter baumannii*. J Bacteriol 204:e0060121. doi:10.1128/jb.00601-21 35191762 PMC8923225

[130] Nguyen B, Ching C, MacGuire A, Casula P, Newman C, Finley F, Godoy VG. 2025. Identification of EppR, a second repressor of error-prone DNA polymerase genes in *Acinetobacter baumannii*. Mol Microbiol 124:20–39. doi:10.1111/mmi.15368 40251897 PMC12242105

[131] Filipek J, Chalaskiewicz K, Kosmider A, Nielipinski M, Michalak A, Bednarkiewicz M, Goslawski-Zeligowski M, Prucnal F, Sekula B, Pietrzyk-Brzezinska AJ. 2024. Comprehensive structural overview of the C-terminal ligand-binding domains of the TetR family regulators. J Struct Biol 216:108071. doi:10.1016/j.jsb.2024.108071 38401830

[132] Corre C. 2013. In search of the missing ligands for TetR family regulators. Chem Biol 20:140–142. doi:10.1016/j.chembiol.2013.02.005 23438741

[133] Cuthbertson L, Nodwell JR. 2013. The TetR family of regulators. Microbiol Mol Biol Rev 77:440–475. doi:10.1128/MMBR.00018-13 24006471 PMC3811609

[134] Cui Q. 2025. Identification and understanding of allostery hotspots in proteins: Integration of deep mutational scanning and multi-faceted computational analyses. J Mol Biol 437:168998. doi:10.1016/j.jmb.2025.168998 39952349 PMC12339756

[135] Dörr T, Lewis K, Vulić M. 2009. SOS response induces persistence to fluoroquinolones in *Escherichia coli*. PLoS Genet 5:e1000760. doi:10.1371/journal.pgen.1000760 20011100 PMC2780357

[136] Liu YJ, Wang X, Sun Y, Feng Y. 2025. Bacterial 5’ UTR: a treasure-trove for post-transcriptional regulation. Biotechnol Adv 78:108478. doi:10.1016/j.biotechadv.2024.108478 39551455

[137] Stagno JR, Wang Y-X. 2024. Riboswitch mechanisms for regulation of P1 helix stability. Int J Mol Sci 25:10682. doi:10.3390/ijms251910682 39409011 PMC11477058

[138] Gozzi K, Ching C, Paruthiyil S, Zhao Y, Godoy-Carter V, Chai Y. 2017. *Bacillus subtilis* utilizes the DNA damage response to manage multicellular development. NPJ Biofilms Microbiomes 3:8. doi:10.1038/s41522-017-0016-3 28649409 PMC5445613

[139] Navidifar T, Amin M, Rashno M. 2019. Effects of sub-inhibitory concentrations of meropenem and tigecycline on the expression of genes regulating pili, efflux pumps and virulence factors involved in biofilm formation by *Acinetobacter baumannii*. Infect Drug Resist 12:1099–1111. doi:10.2147/IDR.S199993 31190904 PMC6512781

[140] Amin M, Navidifar T, Shooshtari FS, Rashno M, Savari M, Jahangirmehr F, Arshadi M. 2019. Association between biofilm formation, structure, and the expression levels of genes related to biofilm formation and biofilm-specific resistance of *Acinetobacter baumannii* strains isolated from burn infection in Ahvaz, Iran. Infect Drug Resist 12:3867–3881. doi:10.2147/IDR.S228981 31853190 PMC6914661

[141] Gurung J, Khyriem AB, Banik A, Lyngdoh WV, Choudhury B, Bhattacharyya P. 2013. Association of biofilm production with multidrug resistance among clinical isolates of *Acinetobacter baumannii* and *Pseudomonas aeruginosa* from intensive care unit. Indian J Crit Care Med 17:214–218. doi:10.4103/0972-5229.118416 24133328 PMC3796899

[142] Li Z, Ding Z, Liu Y, Jin X, Xie J, Li T, Zeng Z, Wang Z, Liu J. 2021. Phenotypic and genotypic characteristics of biofilm formation in clinical isolates of *Acinetobacter baumannii*. Infect Drug Resist 14:2613–2624. doi:10.2147/IDR.S310081 34262306 PMC8274629

[143] Yusef D, Shalakhti T, Awad S, Algharaibeh H, Khasawneh W. 2018. Clinical characteristics and epidemiology of sepsis in the neonatal intensive care unit in the era of multi-drug resistant organisms: a retrospective review. Pediatr Neonatol 59:35–41. doi:10.1016/j.pedneo.2017.06.001 28642139

[144] Pour NK, Dusane DH, Dhakephalkar PK, Zamin FR, Zinjarde SS, Chopade BA. 2011. Biofilm formation by *Acinetobacter baumannii* strains isolated from urinary tract infection and urinary catheters. FEMS Immunol Med Microbiol 62:328–338. doi:10.1111/j.1574-695X.2011.00818.x 21569125

[145] Dijkshoorn L, Nemec A, Seifert H. 2007. An increasing threat in hospitals: multidrug-resistant *Acinetobacter baumannii*. Nat Rev Microbiol 5:939–951. doi:10.1038/nrmicro1789 18007677

[146] Guo S, Vance TDR, Stevens CA, Voets I, Davies PL. 2019. RTX adhesins are key bacterial surface megaproteins in the formation of biofilms. Trends Microbiol 27:453–467. doi:10.1016/j.tim.2018.12.003 30658900

[147] Sharma S, Mohler J, Mahajan SD, Schwartz SA, Bruggemann L, Aalinkeel R. 2023. Microbial biofilm: a review on formation, infection, antibiotic resistance, control measures, and innovative treatment. Microorganisms 11:1614. doi:10.3390/microorganisms11061614 37375116 PMC10305407

[148] Berne C. 2023. Sticky decisions: the multilayered regulation of adhesin production by bacteria. PLoS Genet 19:e1010648. doi:10.1371/journal.pgen.1010648 36862629 PMC9980756

[149] Stones DH, Krachler AM. 2015. Fatal attraction: how bacterial adhesins affect host signaling and what we can learn from them. Int J Mol Sci 16:2626–2640. doi:10.3390/ijms16022626 25625516 PMC4346855

[150] Qin H, Lo NW-S, Loo J, Lin X, Yim AK-Y, Tsui SK-W, Lau TC-K, Ip M, Chan T-F. 2018. Comparative transcriptomics of multidrug-resistant *Acinetobacter baumannii* in response to antibiotic treatments. Sci Rep 8:3515. doi:10.1038/s41598-018-21841-9 29476162 PMC5824817

[151] Walter BM, Cartman ST, Minton NP, Butala M, Rupnik M. 2015. The SOS response master regulator LexA is associated with sporulation, motility and biofilm formation in clostridium difficile. PLoS One 10:e0144763. doi:10.1371/journal.pone.0144763 26682547 PMC4689574

[152] Gotoh H, Kasaraneni N, Devineni N, Dallo SF, Weitao T. 2010. SOS involvement in stress-inducible biofilm formation. Biofouling 26:603–611. doi:10.1080/08927014.2010.501895 20603726

[153] Gotoh H, Zhang Y, Dallo SF, Hong S, Kasaraneni N, Weitao T. 2008. *Pseudomonas aeruginosa*, under DNA replication inhibition, tends to form biofilms via Arr. Res Microbiol 159:294–302. doi:10.1016/j.resmic.2008.02.002 18434096

[154] Bernier SP, Lebeaux D, DeFrancesco AS, Valomon A, Soubigou G, Coppée J-Y, Ghigo J-M, Beloin C. 2013. Starvation, together with the SOS response, mediates high biofilm-specific tolerance to the fluoroquinolone ofloxacin. PLoS Genet 9:e1003144. doi:10.1371/journal.pgen.1003144 23300476 PMC3536669

[155] Costa SB, Campos ACC, Pereira ACM, de Mattos-Guaraldi AL, Júnior RH, Rosa ACP, Asad LMBO. 2014. Adherence to abiotic surface induces SOS response in *Escherichia coli* K-12 strains under aerobic and anaerobic conditions. Microbiology (Reading) 160:1964–1973. doi:10.1099/mic.0.075317-0 25012969

[156] Shortridge MD, Triplet T, Revesz P, Griep MA, Powers R. 2011. Bacterial protein structures reveal phylum dependent divergence. Comput Biol Chem 35:24–33. doi:10.1016/j.compbiolchem.2010.12.004 21315656 PMC3049983

[157] Coleman GA, Davín AA, Mahendrarajah TA, Szánthó LL, Spang A, Hugenholtz P, Szöllősi GJ, Williams TA. 2021. A rooted phylogeny resolves early bacterial evolution. Science 372:eabe0511. doi:10.1126/science.abe0511 33958449

[158] Miller MC, Resnick JB, Smith BT, Lovett CM Jr. 1996. The *Bacillus subtilis dinR* gene codes for the analogue of *Escherichia coli* LexA. J Biol Chem 271:33502–33508. doi:10.1074/jbc.271.52.33502 8969214

[159] Lovett CM, Love PE, Yasbin RE, Roberts JW. 1988. SOS-like induction in *Bacillus subtilis*: induction of the RecA protein analog and a damage-inducible operon by DNA damage in Rec+ and DNA repair-deficient strains. J Bacteriol 170:1467–1474. doi:10.1128/jb.170.4.1467-1474.1988 3127374 PMC210990

[160] Godoy VG, Jarosz DF, Simon SM, Abyzov A, Ilyin V, Walker GC. 2007. UmuD and RecA directly modulate the mutagenic potential of the Y family DNA polymerase DinB. Mol Cell 28:1058–1070. doi:10.1016/j.molcel.2007.10.025 18158902 PMC2265384

[161] Yahya AH, Harston SR, Colton WL, Cabeen MT. 2023. Distinct screening approaches uncover PA14_36820 and RecA as negative regulators of biofilm phenotypes in *Pseudomonas aeruginosa* PA14. Microbiol Spectr 11:e0377422. doi:10.1128/spectrum.03774-22 36971546 PMC10100956

[162] Kidane D, Graumann PL. 2005. Intracellular protein and DNA dynamics in competent *Bacillus subtilis* cells. Cell 122:73–84. doi:10.1016/j.cell.2005.04.036 16009134

[163] Karlin S, Brocchieri L. 1996. Evolutionary conservation of RecA genes in relation to protein structure and function. J Bacteriol 178:1881–1894. doi:10.1128/jb.178.7.1881-1894.1996 8606161 PMC177882

[164] Rocha EPC, Cornet E, Michel B. 2005. Comparative and evolutionary analysis of the bacterial homologous recombination systems. PLoS Genet 1:e15. doi:10.1371/journal.pgen.0010015 16132081 PMC1193525

[165] Nguyen B, Soo MW, Tierney GA, Amorelli EA, Herlihy AL, Godoy VG. 2025. A new component of the DNA damage response biofilm axis is a TetR-like DNA damage response regulator in *Acinetobacter baumannii*. bioRxiv. doi:10.1101/2025.06.05.658052 PMC13078170 41874431

[166] Nicole TC, Alicyn RP, Elliot I, Elizabeth A, Austen H, Nathan T, Matteo CF, Marcello T, Carlos R, Yunrong C, Veronica G-C. 2025. Understanding the genetic diversity of bacteria isolated from across the atacama desert. Ecol Divers 2:10014–10014. doi:10.70322/ecoldivers.2025.10014

[167] Reverdy A, Hathaway D, Jha J, Michaels G, Sullivan J, McAdoo DD, Riquelme C, Chai Y, Godoy-Carter V. 2024. Insights into the diversity and survival strategies of soil bacterial isolates from the Atacama Desert. Front Microbiol 15:1335989. doi:10.3389/fmicb.2024.1335989 38516016 PMC10955380

[168] Alexandre G. 2025. Movement of bacteria in the soil and the rhizosphere. Appl Environ Microbiol 91:e0024625. doi:10.1128/aem.00246-25 40938096 PMC12542687

